# Antibody testing for COVID-19 in patients with acute coronary syndrome in Aichi Prefecture

**DOI:** 10.20407/fmj.2020-032

**Published:** 2021-08-20

**Authors:** Masataka Yoshinaga, Takashi Muramatsu, Hidetsugu Fujigaki, Kuniaki Saito, Hideo Izawa

**Affiliations:** 1 Department of Cardiology, Fujita Health University, School of Medicine, Toyoake, Aichi, Japan; 2 Department of Disease Control and Prevention, Fujita Health University, Graduate School of Health Sciences, Toyoake, Aichi, Japan

**Keywords:** COVID-19, Japan, Acute coronary syndrome, Antibody, Screening

The currently ongoing coronavirus disease 2019 (COVID-19) pandemic was officially recognized as a worldwide pandemic by the World Health Organization (WHO) on March 11, 2020. On April 16, 2020, the Japanese government declared a state of emergency throughout Japan.

Previous work has suggested that COVID-19 may be associated with an elevated risk of systemic thrombotic diseases owing to the inflammatory responses, endothelial dysfunction, and hypercoagulability associated with this disease.^1^ Evidence of myocardial injury (i.e., an elevated level of cardiac troponin) is often observed among hospitalized patients with COVID-19; however, the COVID-19 prevalence among patients with acute coronary syndrome (ACS) has not yet been investigated.

Current clinical practice guidelines strongly recommend diagnostic catheterization and early revascularization (e.g., percutaneous coronary intervention [PCI]) if a patient is considered to have either ST-segment elevation myocardial infarction or high-risk non-ST-segment elevation ACS. Given the potential risk of infectious disease transmission among healthcare providers in the setting of ACS, which potentially requires intensive care and/or emergent catheterization procedures, quick and reliable screening tests are desirable. A Japanese nationwide survey reported that the majority of COVID-19 screening measures applied to such patients included physical examination only (~50%–80%), followed by chest computed tomography (CT) (~10%–50%), and polymerase chain reaction (PCR) (<10%).^2^ Notably, because PCR testing for severe acute respiratory syndrome coronavirus 2 (SARS-CoV-2) is performed less frequently in Japan than in other countries, a considerably larger number of patients undergoing COVID-19 screening have an available CT scan. However, the Centers for Disease Control (CDC) guidelines recommend against using chest CT alone for the diagnosis of COVID-19.

The level of serum antiviral antibodies in patients who have recovered from SARS-CoV-2 infection does not reduce until 4 months after the initial diagnosis.^3^ The fully automated immunoassay system VITROS^®^ (Ortho Clinical Diagnostics, Raritan, NJ, USA) is a novel approach for qualitative serologic testing for antibodies against SARS-CoV-2, the causative pathogen of COVID-19. The precision of the VITROS^®^ anti-SARS-CoV-2 total assay and its cross-reactivity with other acute respiratory virus infections were clinically validated in previous work.^4^ Using the PCR method as a standard, the sensitivity, specificity, positive predictive value, negative predictive value, and accuracy of the antibody test were 71% (56/79), 100% (57/57), 100% (56/56), 71% (57/80), and 83% (113/136), respectively. These data suggest that antibody testing performed with this system could be a useful tool with which to screen for previous exposure to SARS-CoV-2.

In the present study, we employed the VITROS^®^ system to examine the paired IgG, IgM, and IgA against SARS-CoV-2 in 35 consecutive patients who presented with ACS and required PCI during the period from August 17, 2020 through September 30, 2020. This study was conducted in a prospective, single-center, observational fashion (Fujita Health University: HM20-181).

The subjects were aged 69±13 (mean±SD) years, and 62% of them were male. Clinical presentation of ACS on admission was either ST-segment elevation myocardial infarction (23 patients, 66%) or non-ST-segment elevation ACS (12 patients, 34%). At the time of admission, six (17%) of the patients had been prescribed an antithrombotic drug (i.e., low-dose aspirin). The in-hospital mortality rate was 11% (4 patients). None of the patients tested positive for SARS-CoV-2-specific antibodies during their hospitalization.

A major limitation of this study is the small number of subjects tested; however, our data suggest that the prevalence of COVID-19 among ACS patients in Aichi Prefecture or throughout Japan may not be particularly high. Therefore, our results may encourage clinicians to continue conducting diagnostic and treatment procedures for ACS patients, still with careful precautions, even during the ongoing COVID-19 pandemic.
